# Peri-articular administration of tranexamic acid is an alternative route in total knee arthroplasty: a systematic review and meta-analysis

**DOI:** 10.1186/s13018-022-03095-4

**Published:** 2022-04-07

**Authors:** DingYuan Fan, Jia Ma, XiaoHua Liu, Lei Zhang

**Affiliations:** 1 Department of Joint Surgery and Sports Medicine, Wangjing Hospital, China Academy of Chinese Medical Sciences, No. 6, South Zhonghuan Road, Chaoyang district, Beijing, 100102 People’s Republic of China; 2 Beijing University of Chinese Medicine (BUCM), Beijing, People’s Republic of China

**Keywords:** Peri-articular, Intravenous, Intra-articular, Tranexamic acid, TXA, Knee arthroplasty

## Abstract

**Background:**

As an antifibrinolytic agent, tranexamic acid (TXA) is increasingly used in total knee arthroplasty (TKA) to reduce blood loss. The administration of intravenous and intra-articular TXA has been well explored, but the most efficient way to administer TXA remains in question. Peri-articular injection (PAI) of TXA is a recently mentioned method. A meta-analysis of the efficacy of PAI TXA in patients after TKA should be performed.

**Methods:**

A systematic search was performed within PubMed, Embase, and the Cochrane Library up to November 8, 2021. Two authors independently screened studies for eligibility and extracted data for analysis. The primary outcome was haemoglobin change. The secondary outcomes were haematocrit change, total drainage volume, thromboembolic events, and blood transfusion.

**Results:**

A total of ten studies were included in this meta-analysis. The results indicated that there was a significant decrease in haemoglobin change when using PAI TXA compared with no TXA (mean difference − 1.05; 95% CI − 1.28 to − 0.81; *P* < 0.00001; *I*^2^ = 0%), but it had no significant differences compared with IA and IV (mean difference − 0.01; 95% CI − 0.17 to − 0.14; *P* = 0.85; *I*^2^ = 39%). There were no significant differences between the TXA < 1.5 g subgroup (0.10, 95% CI − 0.27 to 0.46; *P* = 0.60; *I*^2^ = 0%) and the TXA ≥ 1.5 g subgroup (0.18, 95% CI − 0.12 to 0.48; *P* = 0.24; *I*^2^ = 74%). In addition, the combined group (PAI plus IV or IA) was superior to the IV or IA group in terms of haemoglobin change (mean difference − 0.51; 95% CI − 0.76 to − 0.27; *P* < 0.0001; *I*^2^ = 19%). Regarding haematocrit change, the pooled result showed it was significantly less in the PAI group than the non-TXA group. Similarly, comparing it against the IV subgroup, the result revealed a difference in favour of the PAI group, with a mean difference of − 1.89 g/dL (95% CI − 2.82 to − 0.95; *P* < 0.0001; *I*^2^ = 67%). For total drainage volume, the pooled result was in favour of PAI TXA over no TXA (297 ml, 95% CI − 497.26 to − 97.23; *P* = 0.004; *I*^2^ = 87%), but it had no significant difference compared with IA and IV (mean difference − 37.98; 95% CI − 115.68 to 39.71; *P* = 0.34; *I*^2^ = 95%). There was no significant difference in thromboembolic events (OR 0.74; 95% CI 0.25 to 2.21; *P* = 0.59; *I*^2^ = 0%). Blood transfusion was not significantly different between the PAI group and the non-TXA group (OR 0.50; 95% CI 0.23 to 1.06; *P* = 0.07; *I*^2^ = 21%), and there was no significant difference between PAI and the other two TXA injection methods (OR 0.72; 95% CI 0.41 to 1.25; *P* = 0.24; *I*^2^ = 19%).

**Conclusion:**

PAI has comparable effects to IV and IA injections. PAI is an alternative injection route of TXA for patients who have undergone TKA.

## Introduction

Total knee arthroplasty (TKA) is a safe and reliable surgical procedure for patients with osteoarthritis, rheumatoid arthritis, or fractures [1–3]. Due to the ageing of its population, the annual demand for knee joint replacement continues to grow in the USA [4]. It is important to improve patient safety and satisfaction during and after TKA. Although considerable advances in anaesthetic and surgical techniques have been made, TKA is still associated with much perioperative blood loss [5]. The estimated intraoperative blood loss is between 500 and 1500 ml for total joint arthroplasty [6]. A post-operative haemoglobin decline between 1 and 3 g/dL has also been reported [7]. Tourniquet was used during knee surgical procedures because of its haemostatic function [8, 9]. However, the recent literature that is full of controversy has raised controversy over its use [10–14]. In addition, it may be associated with an increased risk of serious adverse events, pain, and a rising more extended hospital stay [15, 16].

Tranexamic acid (TXA), a synthetic lysine analogue, is a commonly used antifibrinolytic agent that reduces bleeding and the risk of transfusions in TKA [17, 18]. There are several different methods of TXA administration, such as oral, intravenous (IV), intra-articular (IA), and IV combined with IA application. Several meta-analyses have evaluated the efficacy of different TXA administrations [19–21], but the optimal regimen of tranexamic acid administration is still unclear. Recently, peri-articular injection (PAI) of TXA has been mentioned in TKA. Thus, a systematic review and meta‑analysis needs to be conducted to evaluate the efficacy of PAI in patients who have undergone TKA.

The purpose of this meta-analysis was to investigate the efficacy of PAI in TKA. We hypothesized that (1) PAI would reduce blood loss compared with no TXA and (2) PAI of TXA would have different effects than IV and IA injections. The primary outcome was haemoglobin change. The secondary outcomes were haematocrit change, total drainage volume, thromboembolic events, and blood transfusion.

## Method

This study was performed according to the PRISMA (Preferred Reporting Items for Systematic Reviews and Meta-Analyses) statement and Review Manager 5.3 [22].

### Literature search strategy

We searched three electronic medical databases, PubMed, Embase, and Cochrane Library, for articles published until November 8, 2021. We used the following search strategy: (((peri-articular) OR (peri-articular)) OR (peri-articular Injection)) OR (peri-articular Injection) and (tranexamic acid) OR (TXA). No restrictions by language or publication time were employed. We also checked the references of the most relevant articles.

### Inclusion and exclusion criteria

The included studies met the following inclusion criteria:

1. Patients undergoing total knee arthroplasty.
2. Experimental group: PAI of TXA or peri-articular injection of tranexamic acid combined with intravenous injection or intra-articular injection.
3. Control group: intravenous injection of TXA, intra-articular TXA, or no TXA.
4. Outcomes measured: haemoglobin change, haematocrit change, total drainage volume, thromboembolic events, and blood transfusion.
5. Randomized controlled trial (RCT), prospective cohort study, retrospective study.

The exclusion criteria were (1) therapeutic case series; (2) literature reviews; (3) case reports; (4) cadaver studies; and (5) biomechanical studies.

### Selection of studies

Two authors independently applied the selection criteria. Eligibility screening consisted of the following steps: first, titles, abstracts, and methods were screened for meeting the inclusion criteria; then, the full-text was screened for eligibility for this meta-analysis. We resolved disagreements by discussion, and the third author made the final decision.

### Data extraction

Two independent authors extracted data. Any disagreement on data extraction was resolved by the third author. The data were extracted into a data collection sheet, which included author name, title, year of publication, region, age, sex, BMI, study design, TXA administration, transfusion criteria, prothrombin time (PT), activated partial thromboplastin time (APTT), tourniquet time, haemoglobin change, haematocrit change, drainage volume, thromboembolic events, and blood transfusion. When the outcome measures were presented as median and quartiles, we followed McGrath et al.'s method to estimate the mean and standard deviation (SD) [23]. To calculate the net change in measurements (MD), we used the following formula: measure at end of follow-up (post-operative)—measure at baseline (preoperative).

### Risk of bias assessment

Two authors assessed the risk of bias with the Cochrane Collaboration risk-of-bias tool (Version 2.0) for RCTs [24]. The Cochrane Collaboration's tool categorized this risk as “low risk” of bias, “unclear risk” of bias, or “high risk” of bias. The non-randomized clinical studies were assessed by the Newcastle–Ottawa Scale (NOS) [25]. Using the NOS scale, each study is judged on eight items covering three domains: selection, comparability, and outcome. We used the Kappa score to calculate the agreement degree between reviewers [26]. A score of 0–0.20 represents poor agreement; 0.21–0.40 represents fair agreement; 0.41–0.60 represents moderate agreement; 0.61–0.80 represents good agreement; and 0.81–1.00 represents perfect agreement.

### Assessment of methodological quality

We used the modified Coleman methodology score (MCMS) to assess methodological research quality [27]. The MCMS has a scaled possible score ranging from 0 to 100. A score of 85–100 is considered excellent, 70–84 is deemed good, 55–69 is deemed fair, and less than 55 is deemed poor [27].

### Statistical analysis

We performed a meta-analysis if two or more studies reported on the outcome of interest. We tested for heterogeneity among the included studies by the I-square test, which quantifies the variability in effect estimates due to heterogeneity. The I-square test was interpreted according to the recommendations of the Cochrane Handbook of Systematic Reviews and Meta-analysis (0–40%, not important; 30–60%, moderate heterogeneity; 50–90%, substantial heterogeneity; and 75–100%, considerable heterogeneity). We used the fixed-effect model when no significant heterogeneity was present (*I*^2 ^< 50%;). Otherwise, a random-effect model was used. In addition, we used subgroup analysis to compare PAI and other methods of TXA administration and to exclude potential bias by grouping different literature types if the data were available.

Subgroups:

1. Peri-articular injection (PAI) group vs. intravenous (IV) group or intra-articular (IA) group
2. TXA administration < 1.5 g or TXA administration ≥ 1.5 g
3. Randomized controlled study or cohort study

## Results

**Fig. 1:**
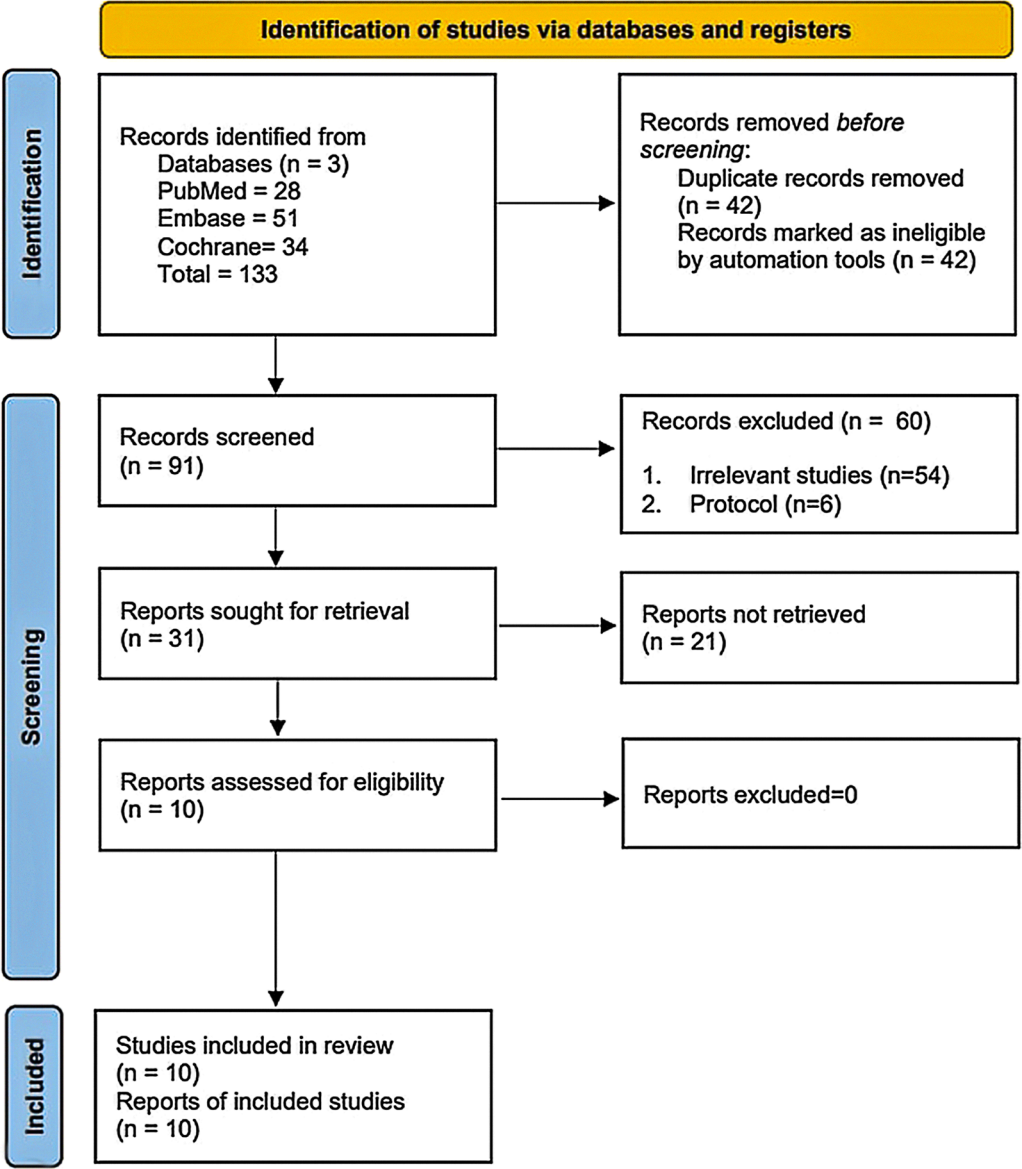
PRISMA flow diagram

In the initial retrieval from the 3 databases, we identified a total of 133 studies, among which 42 studies were duplicate studies excluded by EndNote software (Version X7). After screening the title and abstract, 54 irrelevant studies and six protocols were removed. One case series was removed [28]. Thus, ten studies (5 RCTs, 1 prospective comparative study and 4 retrospective studies) were finally included in the meta-analysis [29–38] (Fig. 1).

### Demographics and characteristics

All studies were published between 2016 and 2021. Four studies were conducted in China [30, 32, 34, 37], two in Thailand [29, 36], and one each in Japan [31], Greece [33], Singapore [35], and Korea [38]. Three studies included a comparison of PAI versus IV injection [29, 37, 38], and six studies included a comparison of PAI versus IA [30, 32–36]. One study only compared PAI versus no TXA injection [31] (Table 1).

**Table 1:** Overview of the included studies

Author	Title	Year	Region	Age	Gender	BMI	Study design
Pinsornsak P	Peri-articular tranexamic acid injection in total knee arthroplasty: a randomized controlled trial	2016	Thailand	PAI 67.63 IV 69.97	PAI 5M/25F IV 7M/23F	PAI 27.96 IV 26.52	A randomized controlled trial
Mao Z	A comparative, retrospective study of peri-articular and intra-articular injection of tranexamic acid for the management of postoperative blood loss after total knee arthroplasty	2016	China	PAI 68.5 IA 69.7 Non-TXA 69.6	PAI 8M/41F IA 5M/31F Non-TXA 10M/32F	PAI 25.9 IA 25.6 Non-TXA 26.6	A retrospective study
Yozawa S	Periarticular injection of tranexamic acid reduces blood loss and the necessity for allogeneic transfusion after total knee arthroplasty using autologous transfusion: a retrospective observational study	2017	Japan	PAI 75.1 Non-TXA 73.0	PAI 13M/31F Non-TXA 10M/34F	PAI 26.4 Non-TXA 26.0	A retrospective study
Zhang S	Multi-route applications of tranexamic acid to reduce blood loss after total knee arthroplasty: a randomized controlled trial	2019	China	PAI 66 IA 68.5 PAI + IA 66 Non-TXA 68	PAI 16M/37F IA 14M/38F PAI + IA 11M/39F Non-TXA 12M/43F	PAI 25.98 IA 25.32 PAI + IA 25.52 Non-TXA 25.28	A randomized controlled trial
Besiris GT	Topical use of tranexamic acid in primary total knee arthroplasty: a comparative study	2020	Greece	PAI 72.08 IA 72.27	NR	NR	A observational prospective comparative study
Lin YK	Significantly reducing blood loss via a peri-articular injection of tranexamic acid during total knee arthroplasty: a retrospective study	2021	China	70.46	18M/32F	27.65	A retrospective study
Sivasubramanian H	Local infiltration of analgesia and tranexamic acid is safe and efficacious in reducing blood loss and comparable to intra-articular tranexamic acid in total knee replacements	2021	Singapore	PAI 65.5 IA 66.8	PAI 9M/12F IA 20M/22F	PAI 20.6 IA 21.5	A retrospective study
Pinsornsak P	Efficacy and systemic absorption of peri-articular versus intra-articular administration of tranexamic acid in total knee arthroplasty: a prospective randomized controlled trial	2021	Thailand	PAI 65.6 IA 68.4 Non-TXA 68.6	PAI 2M/34F IA 3M/33F Non-TXA 4M/32F	PAI 27.4 IA 26.9 Non-TXA 25.6	A prospective randomized controlled trial
Peng HM	Multimodal Peri-articular Injection with Tranexamic Acid can reduce postoperative blood loss versus intravenous tranexamic acid in total knee arthroplasty: a randomized controlled trial	2021	China	PAI 68.65 IV 68.13	PAI 7M/39F IV 6M/41F	PAI 26.81 IV 27.06	A randomized controlled trial
Kim KI	Tranexamic acid in a periarticular multimodal cocktail injection for blood management in total knee arthroplasty: a prospective randomized study	2021	Korea	IV 72.08 PAI 72.58 PAI + IV 72.35	IV 13M/67F PA 13M/67F PAI + IV 11M/69F	IV 26.36 PAI 26.54 PAI + IV 26.05	A prospective randomized study

PAI, periarticular injection; IV, intravenous; IA, intra-articular injection; TXA, tranexamic acid; M, male; F, female; NR, not report

The doses of TXA used in the included literature varied, and the method of extra-articular injection was inconsistent. Three studies injected into the medial and lateral capsules and the quadriceps tendon [29, 32, 36]. Mao et al. [30] used 2 g TXA and 80 mL normal saline into the soft tissues around the joint cavity. Yozawa et al. [31] injected the area around the medial and lateral capsule, the quadriceps muscle tendon, and the infrapatellar fat pad. Besiris et al. [33] injected 25 ml TXA dilution at the posterior knee joint capsule and surrounding soft tissues. Lin [34] injected 1 g/10 mL TXA into the rectus femoris, vastus medialis, patella tendon, pes anserinus, and posterior capsule. Kim et al. [38] injected it into the area around the medial, lateral, anterior, and posterior capsule; the quadriceps muscle tendon; and the infrapatellar fat pad just prior to cementation. The transfusion criteria were similar in all included studies except for one study that did not describe them [32]. Only two studies reported PT and APTT [30, 32]. Four studies presented their tourniquet time [32, 36–38] (Table 2).

**Table 2:** Overview of the TXA Administration, Transfusion criteria, prothrombin time, activated partial thromboplastin time, and tourniquet time

Author	TXA Administration	Transfusion criteria	PT	APTT	Tourniquet time
Pinsornsak P	PAI: 750 mg TXA into the medial, lateral capsules and the quadriceps tendon prior to capsular closure and tourniquet deflation IV: 750 mg TXA before tourniquet deflation	Hb < 10 g/dl, anaemia, congestive heart failure, unexplained tachycardia, hypotension unresponsive to fluid replacement	NR	NR	NR
Mao Z	PAI: 2 g TXA and 80 mL normal saline into the soft tissues around the joint cavity, 5 to 10 mL at each point, such as posterior joint capsulae synovial membrane and ligaments, especially the sites of soft tissue release and incisal edges in the synovial membrane IA: 2 g TXA and 80 mL normal saline into the knee joint cavity after wound closure	Hb < 8 g/dl, 8–10 g/dl with hemodynamic instability	PAI 11.5 ± 2.5 IA 11.1 ± 0.7 Non-TXA 11.1 ± 0.6	PAI 27.0 ± 4.3 IA 27.0 ± 3.3 Non-TXA 27.0 ± 2.9	NR
Yozawa S	PAI: 40 ml of 0.25% ropivacaine with 1: 2000 epinephrine containing 1000 mg TXA(25 mg/ml)into injected into the area around the medial and lateral capsule, the quadriceps muscle tendon, and the infrapatellar fat pad prior to capsular closure Non-TXA: 40 ml of 0.25% ropivacaine with 1: 2000 epinephrine	Hb < 8 g/dl	NR	NR	NR
Zhang S	PAI: TXA solution (1 g TXA, 20 ml saline) comprising 5 ml to the medial capsule, 5 ml to the lateral capsule, and10 ml to the soft tissue around quadriceps femoris IA: TXA solution of 20 ml (TXA 1 g + 20 ml saline) was injected into the articular cavity after suture incision Non-TXA: injection of the same amount of saline at the same place	NR	PAI 11.60 (11.20,12.15) IA 11.65 (11.00,12.05) PAI + IA 11.50 (10.98,12.00) Non-TXA 11.50 (11.10,12.00)	PAI 25.40 (22.85,27.50) IA 24.05 (21.63,26.38) PAI + IA 24.25 (22.35,26.75) Non-TXA 24.10 (23.00,27.00)	PAI 50.00 (42.00,62.50) IA 49.50 (40.00,59.75) PAI + IA 53.00 (37.50,63.25) Non-TXA 44.00 (36.00,68.00)
Besiris GT	PAI: 1.5 g TXA diluted in 50 ml of normal saline. Prior to final prostheses placement 25 ml of the dilution were injection at the posterior knee joint capsule. Following the final prostheses placement, the remaining dilution was injection at the surrounding soft tissues for at least 5 min prior to tourniquet deflation IA: 1.5 g of TXA diluted in 50 ml of normal saline was injected into the knee joint after knee capsule closure for at least 5 min prior to tourniquet deflation	Hb < 8 mg/dl or < 10.0 mg/dl with concomitant symptoms of anaemia or anaemia-related organ dysfunction	NR	NR	NR
Lin YK	PAI: 1 g/10 mL TXA into the rectus femoris, vastus medialis, patella tendon, pes anserinus, and posterior capsule IA: 1 g/10 mL TXA	Hb < 8 mg/dl, Hb > 3.0 mg/dl with intolerable symptoms or organ dysfunction	NR	NR	NR
Sivasubramanian H	PAI: 1 g TXA IA: 1 g TXA	Hb < 8.5 g/dl, symptomatic, cardiovascular comorbidities	NR	NR	NR
Pinsornsak P	PAI: 15 mg/kg TXA was mixed with the anaesthetic cocktail and injected into peri-articular soft tissue without posterior capsular infiltration, including the medial gutter, lateral gutter, and quadriceps muscle before capsular closure IA: 2 g TXA	Hb < 8 g/dl, Hb 8–10 g/dl with hemodynamic instability	NR	NR	PAI 77.4 ± 4.1 IA 76.5 ± 4.7 Non-TXA 76.1 ± 4.7
Peng HM	PAI: 1000 mg/10 ml TXA, 110 ml of saline as a placebo IV: 1000 mg TXA (110 ml total volume)	Hb < 8 g/dl or < 10 g/dl with symptomatic anaemia, at high risk of cardiac comorbidities	NR	NR	PAI 82.93 ± 3.21 IV 82.36 ± 4.54
Kim KI	PAI: 1 g of TXA mixed with PAMC (150 mg ropivacaine, 0.3 mg epinephrine, 45 mg ketorolac, 40 mg triamcinolone, 5 mg morphine, 1 g cefotiam, and 60 ml normal saline) injected into the area around the medial, lateral, anterior and posterior capsule; the quadriceps muscle tendon; and the infrapatellar fat pad just prior to cementation IV: a dose of 15 mg/kg TXA with 100 ml normal saline twice in the TKA perioperative period PAI + IV: both IV and PAMC injections	8.0 g/dL with clinical symptoms of anaemia	NR	NR	IV 64.31 ± 6.63 PAI 63.54 ± 5.42 PAI + IV 63.71 ± 5.13

PAI, periarticular injection; PAMC, periarticular multimodal cocktail; IV, intravenous; IA, intra-articular injection; TXA, tranexamic acid; NR, not report; PT, prothrombin time; APTT, activated partial thromboplastin time; TKA, total knee arthroplasty

### Risk of bias

Five RCTs performed adequate random sequence generation and allocation concealment. Only one study had a low risk of performance bias [37]. All RCTs had unclear risks of detection bias, which may have lowered the accuracy of the results. Incomplete outcome reporting, selective reporting, and other biases were at low risk in five RCTs. All papers but one were given seven stars. The Kappa score between the two reviewers was 0.84 (Table 3).

**Table 3:** The Newcastle–Ottawa Scale (NOS)

Author	Selection				Comparability	Outcome			Score
	Representativeness of the exposed cohort	Selection of nonexposed cohort	Ascertainment of exposure	Demonstration that the outcome of interest was not present at the start of the study	Comparability of cohorts on the basis of the design or analysis	Assessment of outcomes	Follow-up was long enough for outcomes to occur	Adequacy of follow-up	
Mao Z	★	★	★	★	★	★	★	–	7
Yozawa S	★	★	★	★	★	★	★	–	7
Besiris GT	★	★	★	★	★	★	★	–	7
Lin YK	★	★	★	★	★	★	★	★	8
Sivasubramanian H	★	★	★	★	★	★	★	–	7

### Methodologic Quality Assessment

**Fig. 2:**
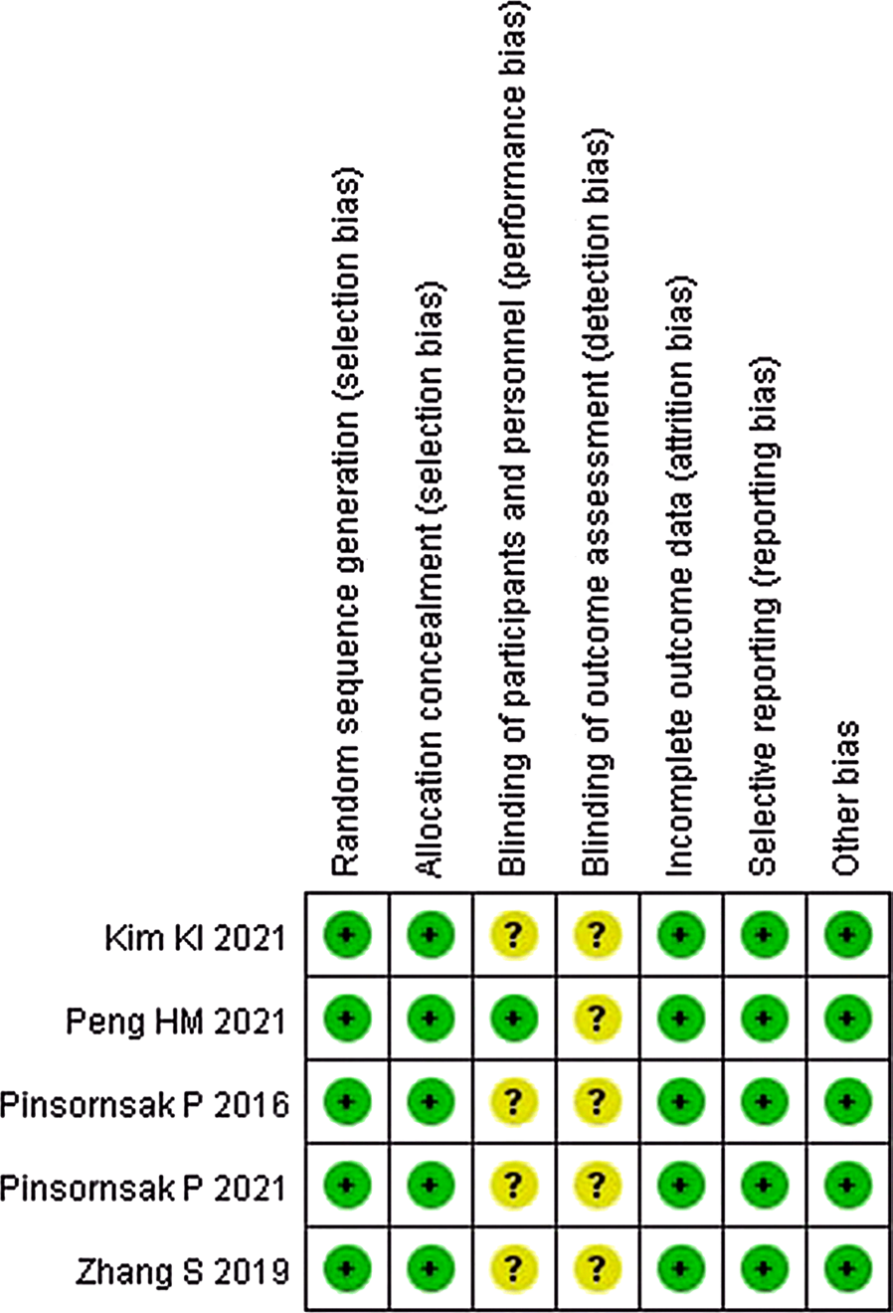
Risk-of-bias summary

There were six good-methodological-quality studies [29, 32, 33, 36–38] and four fair-quality studies [30, 31, 34, 35]. Eight out of ten studies achieved a perfect score on study size [30–33, 35–38]. Only two studies received two points on the follow-up part [31, 38]. All studies achieved an ideal score in surgical procedures. Only one study was given no score for disease diagnosis [31]. Three studies received five points [29, 30, 35], and five studies received three points for the description of the surgical procedure [31, 34, 36–38]. No studies received scores for postoperative rehabilitation. In Part B, all studies achieved a perfect score on outcome criteria. Eight out of ten studies obtained good scores on the procedure to assess outcomes [30–36, 38]. Nine studies achieved a perfect score on the subject selection process [29–38] (Table 4; Fig. 2).

**Table 4:** Modified Coleman Methodology Score (MCMS)

Author	Part A							Part B			
	1. Study size	2. Mean Follow-up	3. Number of different surgical procedures	4. Type of study	5. Diagnostic certainty	6. Description of surgical procedure given	7. Description of postoperative rehabilitation	1. Outcome criteria	2. Procedure to assess outcomes	3. Description of subject selection process	Total score
Pinsornsak P	7	0	10	15	5	5	0	10	15	15	82
Mao Z	10	0	10	0	5	5	0	10	11	15	66
Yozawa S	10	2	10	0	0	3	0	10	11	15	61
Zhang S	10	0	10	15	5	0	0	10	11	15	76
Besiris GT	10	0	10	10	10	0	0	10	11	10	71
Lin YK	7	0	10	0	5	3	0	10	11	15	61
Sivasubramanian H	10	0	10	0	5	5	0	10	11	15	66
Pinsornsak P	10	0	10	15	5	3	0	10	15	15	83
Peng HM	10	0	10	15	5	3	0	10	11	15	79
Kim KI	10	2	10	10	5	3	0	10	11	15	76

### Haemoglobin change

#### PAI vs. non-TXA

**Fig. 3:**
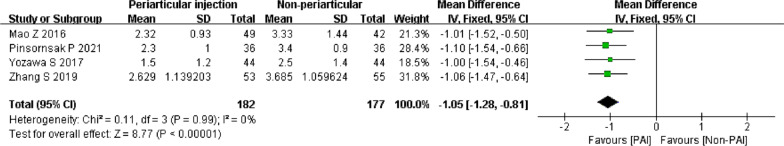
Haemoglobin change, PAI vs. non-TXA

Four studies compared that the PAI group to the non-TXA group on haemoglobin change [30–32, 36]. A total of 359 patients were included in the two groups. The results indicated a significant reduction from using PAI (mean difference − 1.05; 95% CI − 1.28 to − 0.81; *P* < 0.00001; *I*^2^ = 0%) (Fig. 3).

**Fig. 4:**
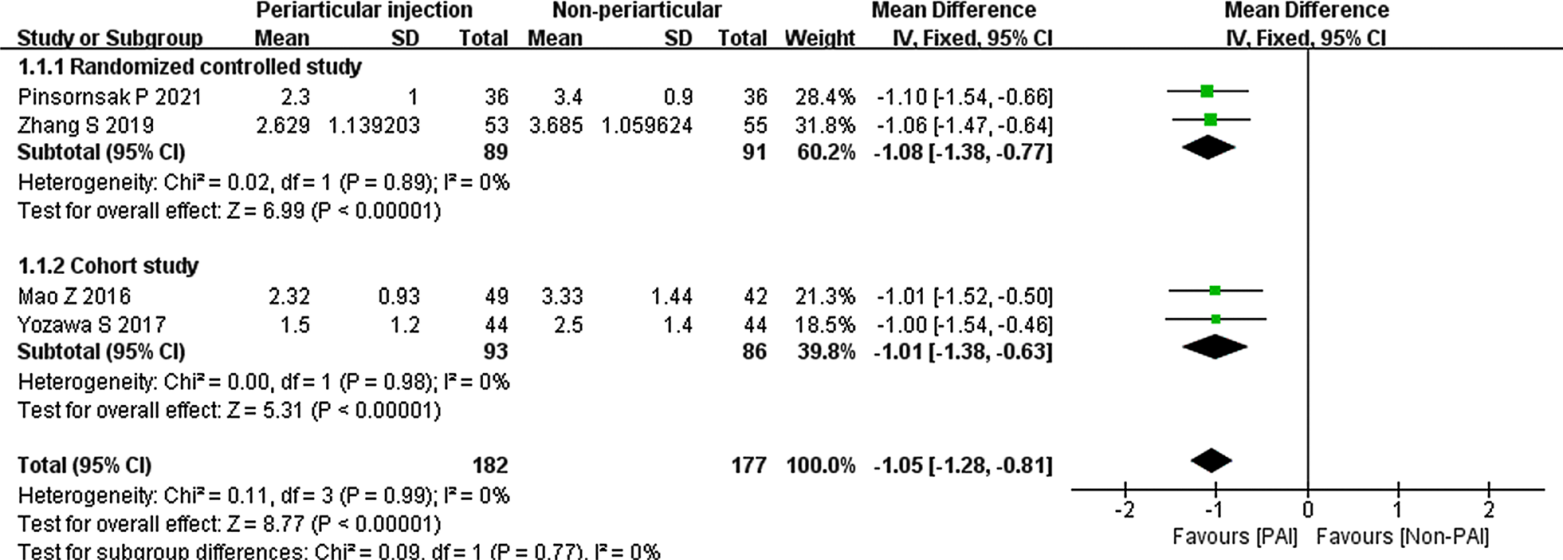
Haemoglobin change, PAI vs. non-TXA subgroup analysis

The subgroup of cohort studies showed similar results. (mean difference − 1.05; 95% CI − 1.28 to − 0.81; *P* < 0.00001; *I*^2^ = 0%) (Fig. 4).

#### PAI vs. IV or IA

**Fig. 5:**
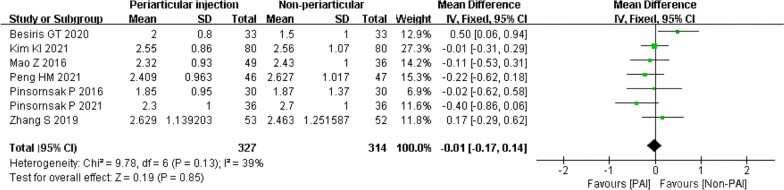
Haemoglobin change, PAI vs. IV or IA

Eight studies compared the PAI group to the IV or IA group on haemoglobin change [29, 30, 32, 33, 35–38]. However, one study expressed the results as the mean without standard deviation [35]. Collectively, the mean difference was − 0.01 (95% CI − 0.17 to 0.14; *P* = 0.85; *I*^2^ = 39%) (Fig. 5).

**Fig. 6:**
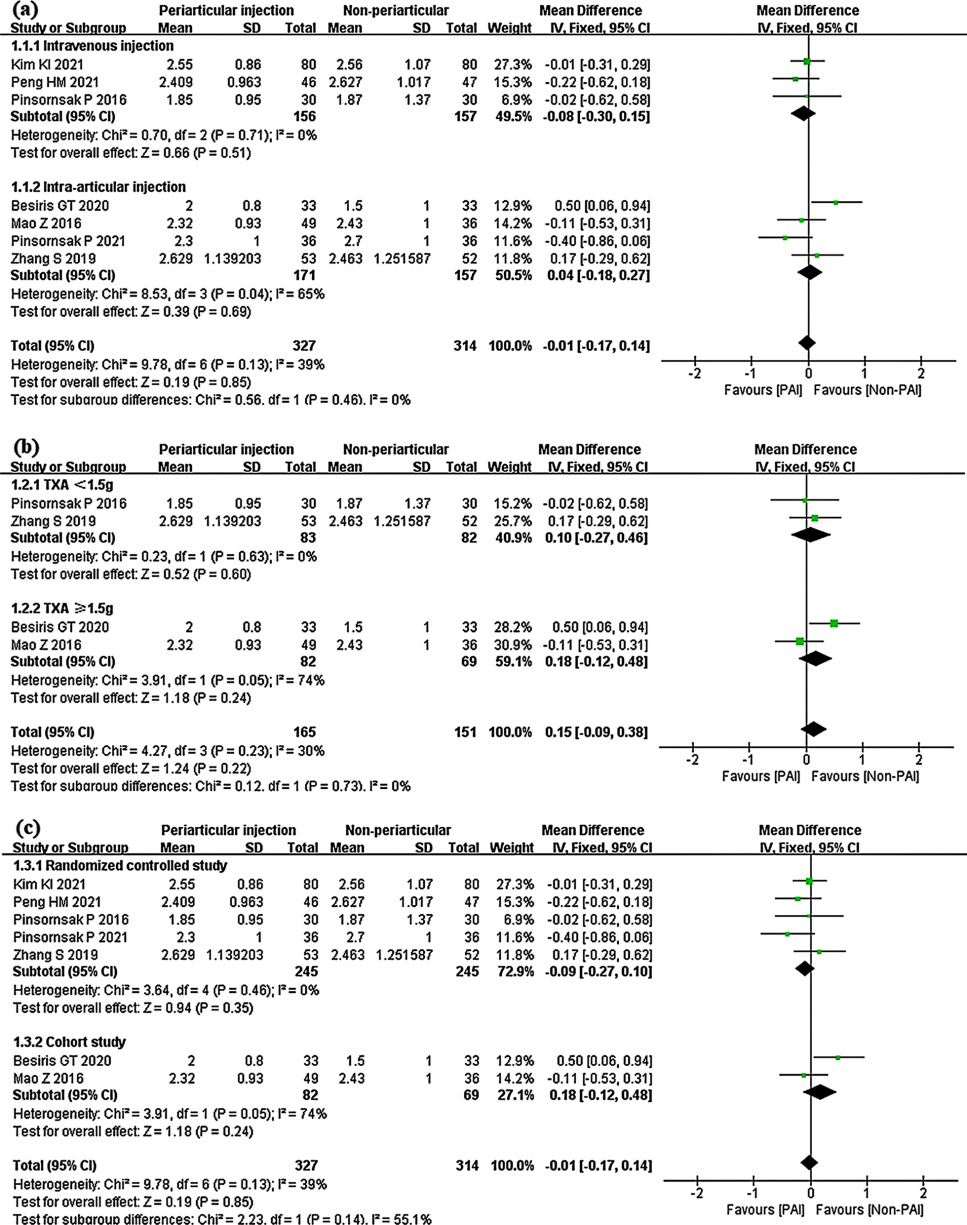
Haemoglobin change, PAI vs. IV or IA subgroup analysis **a** PAI group vs IV group or PAI group vs IA group **b** TXA administration < 1.5 g or TXA administration ≥ 1.5 g **c** Randomized controlled study or cohort study

In the IV subgroup, the PAI showed no significant difference from IV (mean difference − 0.08; 95% CI − 0.30 to 0.15; *P* = 0.51; *I*^2^ = 0%). PAI was also not superior to IA (mean difference 0.04; 95% CI − 0.18 to 0.27; *P* = 0.69; *I*^2^ = 65%). There was no heterogeneity for subgroup differences (Fig. 6a). In the TXA ≤ 1.5 g subgroup, the mean difference was 0.10 (95% CI − 0.27 to 0.46; *P* = 0.60; *I*^2^ = 0%), and in the TXA ≥ 1.5 g subgroup, the mean difference was 0.18 (95% CI − 0.12 to 0.48; *P* = 0.24; *I*^2^ = 74%) (Fig. 6b). The subgroups of randomized controlled studies (mean difference − 0.09; 95% CI − 0.27 to 0.10; *P* = 0.35; *I*^2^ = 0%) and cohort studies (mean difference 0.18; 95% CI − 0.12 to 0.48; *P* = 0.24; *I*^2^ = 74%) showed similar results (Fig. 6c).

#### PAI combined with IV or IA vs. IV or IA alone

**Fig. 7:**
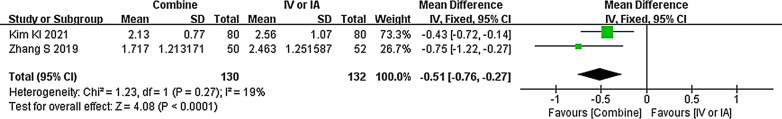
Haemoglobin change, PAI combined with IV or IA vs. IV or IA alone

Only two studies reported PAI combined with IA or IV. The results revealed that the combined group (PAI combined with IV or IA) was superior to the IV or IA group. (mean difference − 0.51; 95% CI − 0.76 to − 0.27; *P* < 0.0001; *I*^2^ = 19%) (Fig. 7).

### Haematocrit change

#### PAI vs. non-TXA

**Fig. 8:**
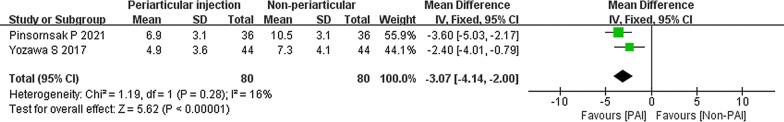
Haematocrit change, PAI vs. non-TXA

Only three studies reported haematocrit changes in the PAI and non-TXA groups [31, 32, 36]. However, the measurement units of haematocrit in one study [32] were inconsistent with those in the other two studies [31, 36]. The pooled mean difference was − 3.07 g/dL in favour of PAI (95% CI − 4.14 to − 2.00; *P* < 00,001; *I*^2^ = 16%) (Fig. 8).

#### PAI vs. IV or IA

**Fig. 9:**
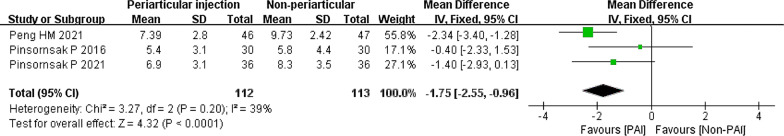
Haematocrit change, PAI vs. IV or IA

Four studies compared the PAI group to the IV or IA group on haemoglobin change [29, 35–37]. However, one study expressed the results as the mean without standard deviation [35]. Collectively, PAI showed a significant reduction in haematocrit change, with a mean difference of − 1.75 g/dL (95% CI − 2.55 to − 0.96; *P* < 0.0001; *I*^2^ = 39%). There was no heterogeneity for subgroup differences (Fig. 9).

**Fig. 10:**
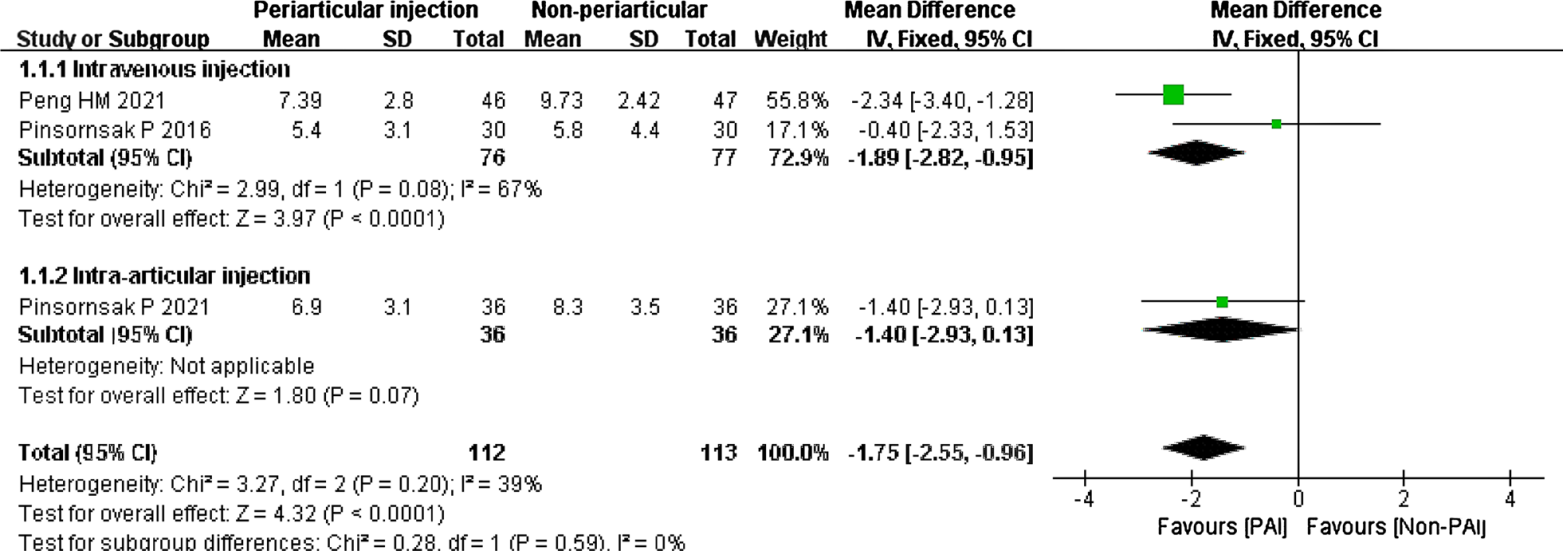
Haematocrit change, PAI vs. IV or IA subgroup analysis

In the IV subgroup, the results revealed a difference in favour of the PAI group, with a mean difference of − 1.89 g/dL (95% CI − 2.82 to − 0.95; *P* < 0.0001; *I*^2^ = 67%) (Fig. 10).

### Total drainage volume

#### PAI vs. non-TXA

**Fig. 11:**
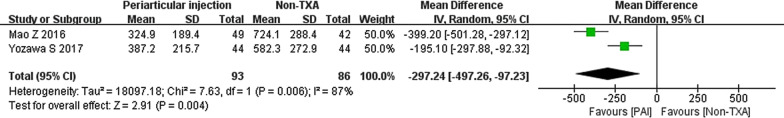
Total drainage volume, PAI vs. non-TXA

Two studies compared the PAI group to the non-TXA group in terms of total drainage volume [30, 31]. The pooled result (mean difference − 297.24 ml 95% CI − 497.26 to − 97.23; *P* = 0.004; *I*^2^ = 87%) indicated a significant reduction in the PAI group (Fig. 11).

#### PAI vs. IV or IA

**Fig. 12:**
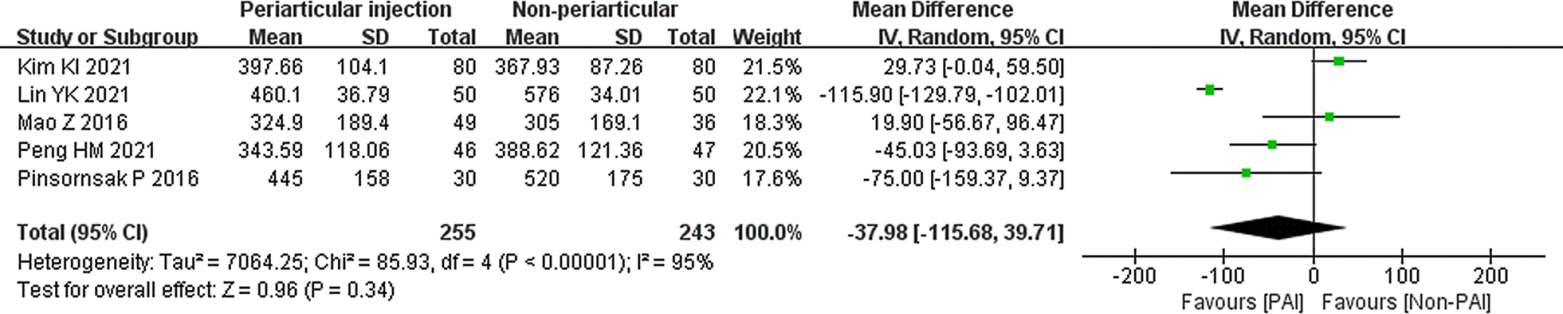
Total drainage volume, PAI vs. IV or IA

Five studies compared the PAI group to the IV or IA group in drainage volume [29, 30, 34, 37, 38]. There were 255 patients in the PAI group, who showed no significant difference from the 243 patients in the non-PAI group (mean difference − 37.98; 95% CI − 155.68 to 39.71; p = 0.34; *I*^2^ = 95%) (Fig. 12).

**Fig. 13:**
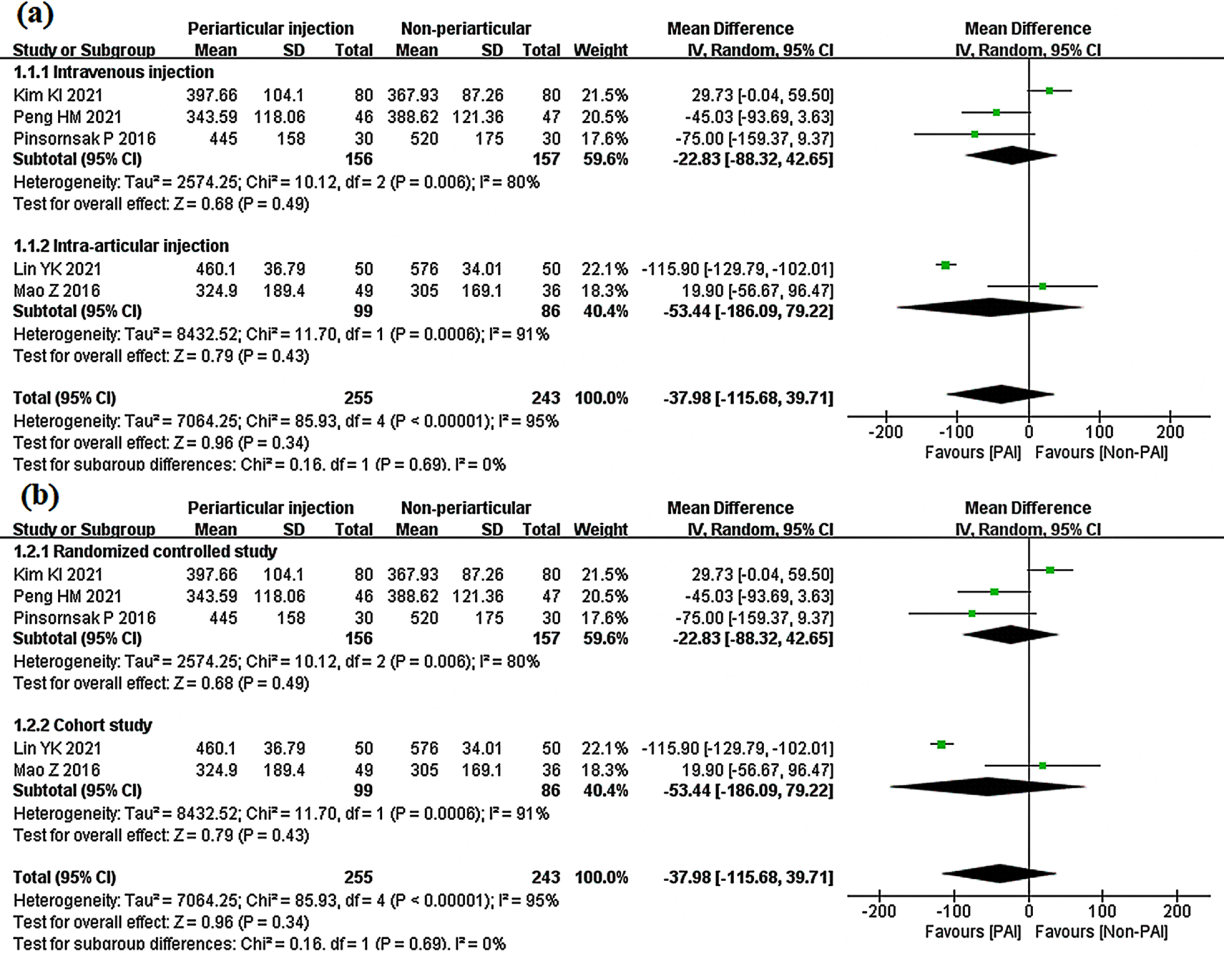
Total drainage volume, PAI vs. IV or IA subgroup analysis **a** PAI group vs IV group or PAI group vs IA group **b** Randomized controlled study or cohort study

In the IV subgroup, the 156 patients in the PAI group showed no significant difference from the 157 patients in the IV group (mean difference − 22.83 ml; 95% CI − 88.32 to 42.65; *P* = 0.49; *I*^2^ = 80%). In the IA subgroup, the pooled result indicated no significant reduction from PAI (mean difference − 53.44 ml 95% CI − 186.09 to 79.22; *P* = 0006; *I*^2^ = 91%). There was no heterogeneity for subgroup differences (Fig. 13a). The subgroups of randomized controlled studies (mean difference − 22.83; 95% CI − 88.32 to 42.65; *P* = 0.49; *I*^2^ = 80%) and cohort studies (mean difference − 53.44; 95% CI − 186.09 to 79.22; *P* = 0.43; *I*^2^ = 91%) showed similar results (Fig. 13b).

### Thromboembolic events

#### PAI vs. IV or IA

**Fig. 14:**
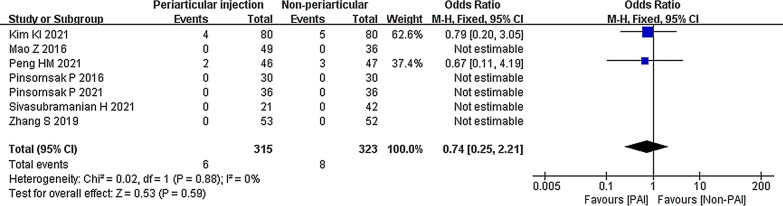
Thromboembolic events, PAI vs. IV or IA

Seven studies compared the PAI group to the IV or IA group on thromboembolic events [29, 30, 32, 35–38]. There was no significant difference between the groups (OR 0.74; 95% CI 0.25 to 2.21; *P* = 0.59; *I*^2^ = 0%) (Fig. 14).

**Fig. 15:**
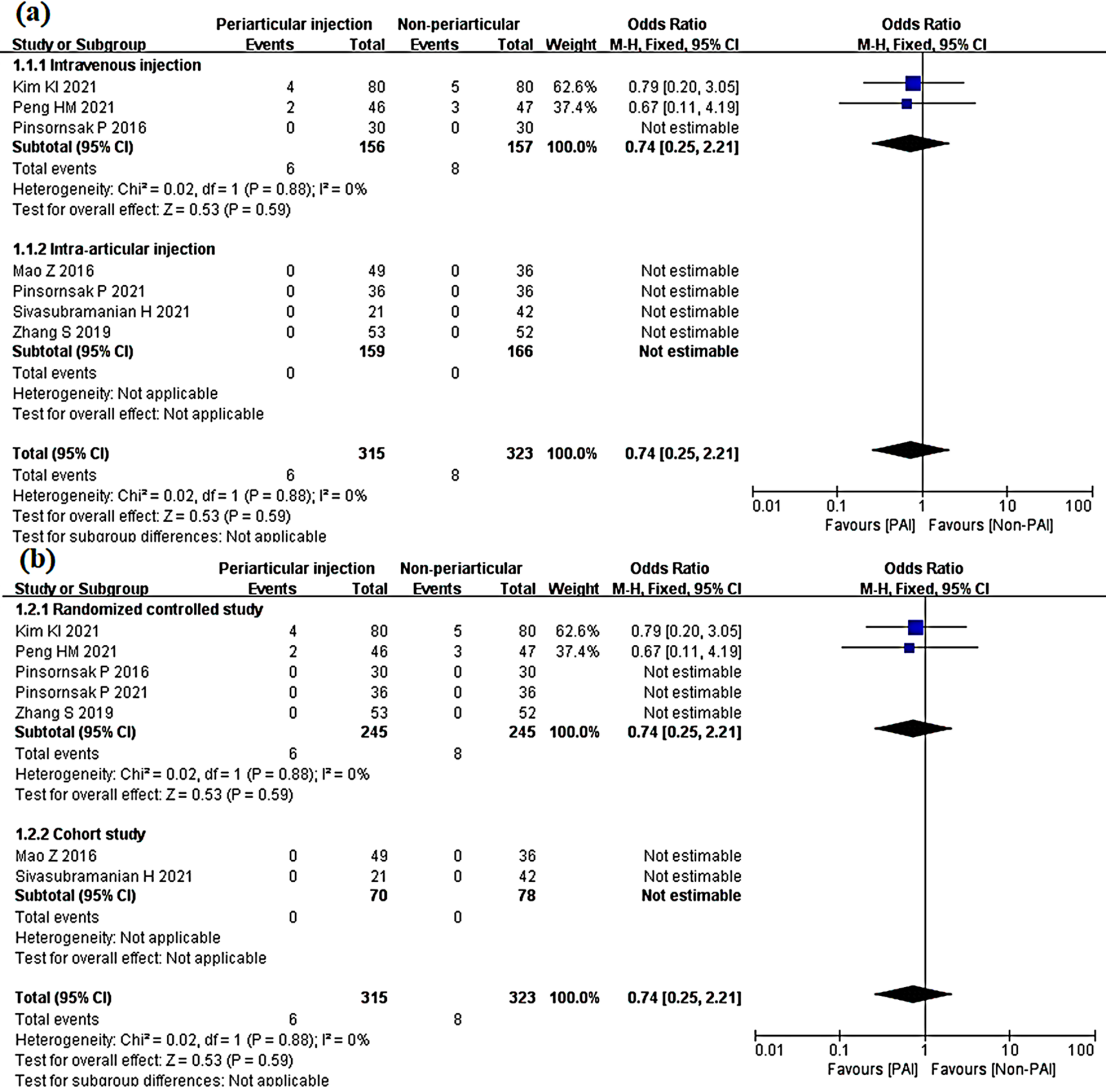
Thromboembolic events, PAI vs. IV or IA subgroup analysis **a** PAI group vs IV group or PAI group vs IA group **b** Randomized controlled study or cohort study

The IV subgroup showed no significant difference from the PAI group (OR 0.74; 95% CI 0.25 to 2.21; *P* = 0.59; *I*^2^ = 0%) (Fig. 15a). A similar result was shown in the randomized controlled study subgroup (OR 0.74; 95% CI 0.25 to 2.21; *P* = 0.59; *I*^2^ = 0%) (Fig. 15b).

#### PAI combined with IV or IA vs. IV or IA alone

**Fig. 16:**
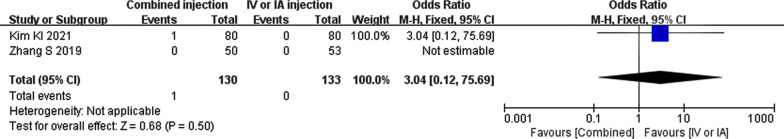
Thromboembolic events, PAI combined with IV or IA vs. IV or IA alone

Only two studies reported on thromboembolic events after PAI combined with IA or IV. The mean difference in the pooled results was 3.04 (95% CI 0.12 to 75.69) (Fig. 16).

### Blood transfusion

#### PAI vs. non-TXA

**Fig. 17:**
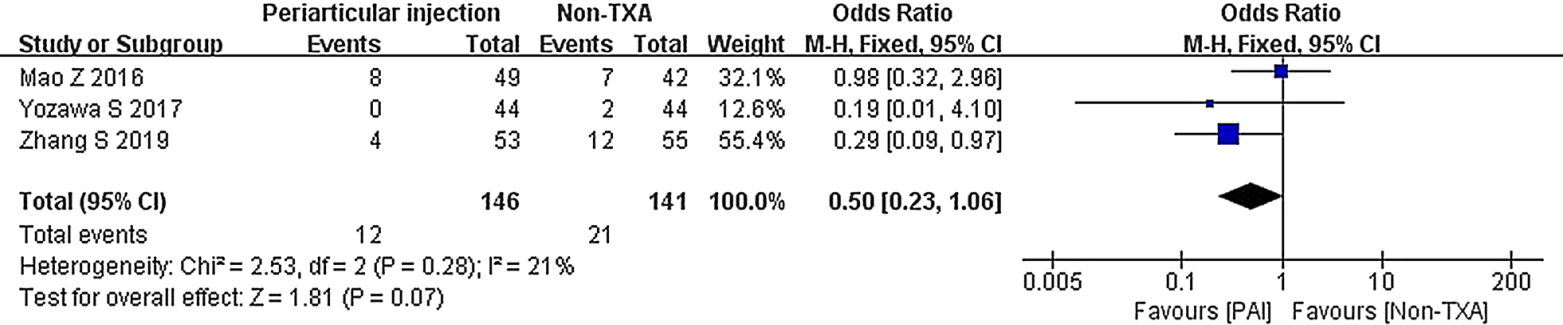
Blood transfusion, PAI vs. non-TXA

Three studies compared the PAI group to the non-TXA group regarding the transfusion rate [30–32]. There was no significant difference between the groups (OR 0.50; 95% CI 0.23 to 1.06; *P* = 0.07; *I*^2^ = 21%) (Fig. 17).

**Fig. 18:**
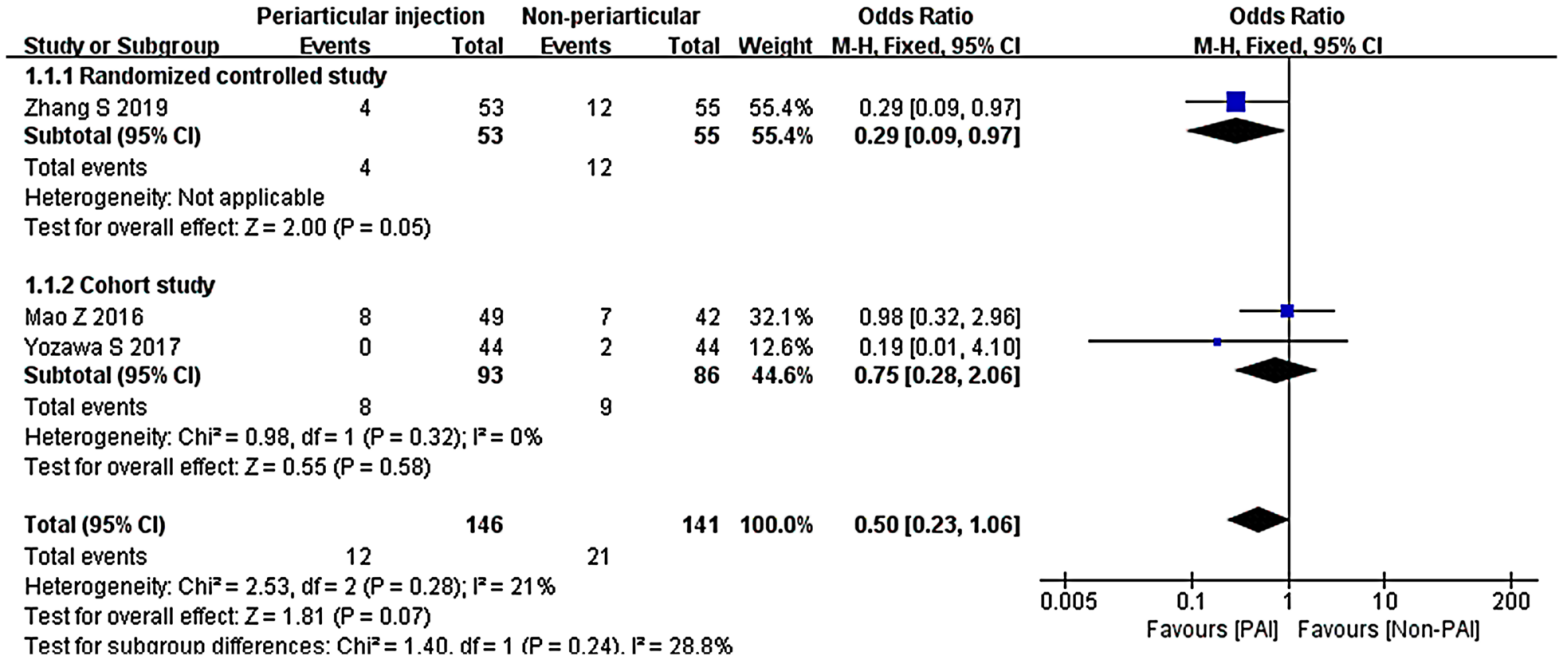
Blood transfusion, PAI vs. non-TXA subgroup analysis

The subgroup of cohort studies (OR 0.75; 95% CI 0.28 to 2.06; *P* = 0.58; *I*^2^ = 0%) showed similar results (Fig. 18).

#### PAI vs. IV or IA

**Fig. 19:**
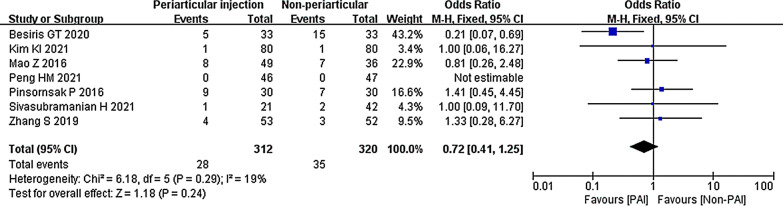
Blood transfusion, PAI vs. IV or IA

Seven studies compared the PAI group with the IV or IA group on transfusion rate [29, 30, 32, 33, 35, 37, 38]. Collectively, there was no significant difference between PAI and the other two TXA injection methods (OR 0.72; 95% CI 0.41 to 1.25; *P* = 0.24; *I*^2^ = 19%). The test for subgroup differences of heterogeneity yielded a value of 46.4% (Fig. 19).

**Fig. 20:**
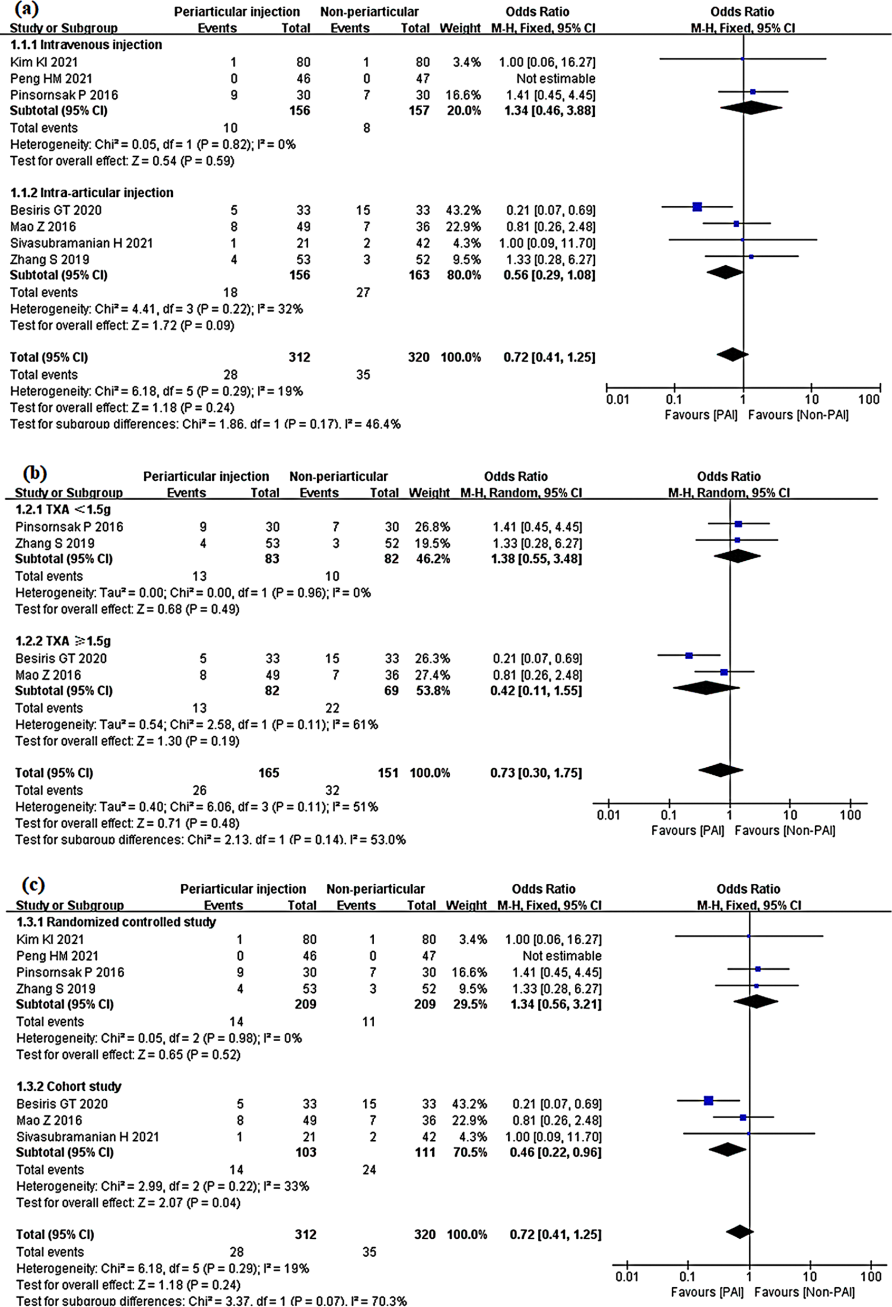
Blood transfusion, PAI vs. IV or IA subgroup analysis **a** PAI group vs IV group or PAI group vs IA group **b** TXA administration < 1.5 g or TXA administration ≥ 1.5 g **c** Randomized controlled study or cohort study

In the IV subgroup, there was no significant difference with the PAI group. (OR 1.34; 95% CI 0.46 to 3.88; *P* = 0.59; *I*^2^ = 0%). Similarly, PAI showed no significant difference from the IA group (OR 0.56; 95% CI 0.29 to 1.08) (Fig. 20a). In the randomized controlled study subgroup, there was no significant difference between the two groups (OR 1.34; 95% CI 0.56 to 3.21; *P* = 0.52; *I*^2^ = 0%). However, in the cohort study subgroup, the PAI group had a lower transfusion rate than the non-PAI group (OR 0.46; 95% CI 0.22 to 0.96; *P* = 0.04; *I*^2^ = 33%) (Fig. 20b).

## Discussion

**Fig. 21:**
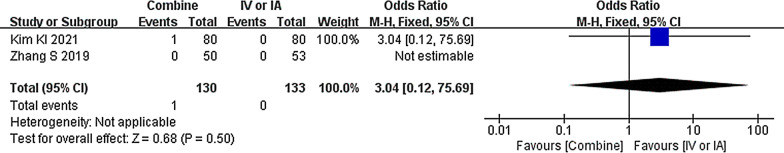
Blood transfusion, PAI combined with IV or IA vs. IV or IA alone

To the best of our knowledge, this study is the first systematic review and meta-analysis of TXA extra-articular injection methods. The results showed that PAI significantly improved postoperative bleeding compared with non-TXA. More importantly, our study found no significant difference between PAI and IV or IA in haemoglobin change, total drainage volume, thromboembolic events, or blood transfusion. When injecting TXA with PAI, there was no significant difference between a dose less than 1.5 g and a dose equal to or greater than 1.5 g. In addition, when PAI was combined with IV or IA, it was superior to the IV or IA group in terms of haemoglobin change (Fig. 21).

TXA, as an antifibrinolytic agent blocking the lysine binding site of plasminogen, can effectively reduce the duration and quantity of blood loss and has been used in orthopaedic surgery [39–41]. Various routes of TXA administration have been used in surgical practice. IV and IA are the two common routes in joint surgery. IV administration requires systemic distribution to exert its antibleeding effects. Concern remains about the safety, as it may cause systemic toxicity, such as thrombosis, acute renal impairment, and systemic hypersensitivity reactions [29, 42, 43]. In addition, the maximum plasma level time of TXA after IV injection has been reported to be 5–15 min [44–46]. IA administration has an insufficient, limited duration of contact to immerse the anterior tissues of the knee joint where the use of post-operative drains is required [29, 30, 35]. During TKA, soft tissue needs to be released to balance knee tension, which may cause the TXA solution to leak or drain out of the joint [30, 31]. Thus, a higher volume or dose of TXA and cost may be needed with IA. PAI is an alternative route for TXA that injects the solution into the soft tissue around the knee joint cavity [29, 30]. Unlike with IA and IV, the surgeon can inject directly into vulnerable bleeding sites for a longer duration [29, 30]. In addition, PAI does not principally limit the use of drainage [31]. Another common concept in arthroplasty is the use of combined IV and IA tranexamic acid. Some studies have reported that combined administration of IV and IA TXA is associated with lower total blood loss, drainage volume, and maximum haemoglobin drop [19, 47–49]. Considering that PAI has similar effects as IV and IA, IV combined with IA may also perform better than PAI in blood management. However, little is known about the safety of intravenous administration in patients with a history of venous thromboembolism, myocardial infarction, cerebrovascular accident, transient ischaemic attack, and stent implantation [50]. PAI is a new method that can reduce the risk to some extent, and in our analysis, it is better when combined with IV or IA than when given alone. Therefore, it can be used as an alternative to IA combined with IV to produce a similar effect.

One issue that needs to be considered is TXA toxicity in human peri-articular tissues. Unlike the previous routes of TKA, PAI directly exposes cartilage, tendons, infrapatellar fat pads, and ligaments to TXA. In the current orthopaedic practice, the interaction between these critical tissues and TXA remains largely unclear [51].

In an experimental study of male rats, 1 ml of locally administered TXA had an adverse effect on tendon healing after six weeks [52]. By contrast, a rat model study investigating the effects of TXA by histopathology and immunohistochemistry showed that TXA did not impair tendon healing [53]. In addition, TXA stimulated TNF-α and MMP-3 expression, as a positive effect in the early period of tendon healing [54]. Similarly, in an experimental rat model, TXA accelerated early bone formation and fracture healing of closed femoral fractures [55]. Ambra LF et al. found that current TXA topical protocols (1, 2, and 4 mg/ml in saline solution) did not present any cytotoxic effects on cartilage explants in a pig model [56]. Similar results of IA TXA administration were supported by Birisik et al. [57]. Their in vitro results suggested that surgeons need to pay attention to the dose of TXA when using PAI. Parker JD et al. found that TXA offered cellular protection for concentrations below 20 mg/ml. Concentrations over 20 mg/ml resulted in atypical morphology, reduced cellular adhesion, and metabolic activity associated with increased chondrocyte death [58]. The dose of 20 mg/mL TXA is a safe limit for topical use [59]. Another study found that toxic effects of TXA occur as early as 2.5 min after exposure, and the threshold dose seems to be 25 mg/ml [60]. Wang et al. found that in 10 min, exposure to 100 mg/ml TXA did not have much of a negative effect on cells. However, chronic exposure to TXA over 25 mg/ml can inhibit viability, proliferation, collagen synthesis, adhesion, and migration and induce apoptosis in fibroblasts [61]. In our study, PAI TXA showed no significant difference in haemoglobin changes when injected at < 1.5 g vs. ≥ 1.5 g. Therefore, for the safety of patients, it is more recommended to use TXA less than 1.5 g. To further reduce blood loss, PAI can be combined with IV and IA.

TXA allergy is another problem that deserves our attention. In 2004, the first case of anaphylactic shock to tranexamic acid was reported during coronary artery bypass graft surgery [62]. TXA allergy has also been reported during orthopaedic surgery. An 80-year-old woman who underwent elective knee replacement developed hypotension, tachycardia, and facial erythema accompanied by increased serum tryptase after intravenous TXA [63]. A 15-year-old male presented a delayed anaphylactic reaction with hypotensive, tachycardic, and erythema during posterior spinal fusion [64]. In 2020, another anaphylactic reaction to TXA during posterior spinal fusion was reported [65]. Although tranexamic acid allergy is rare, prevention measured are still necessary. Li et al. proposed a standardized protocol for TXA anaphylactic reactions. Serial serum tryptase levels (30 to 120 min and 24 h) need to be measured after the reaction. Then, 100 mg/mL, 0.01 mg/mL, and 0.1 mg/mL TXA skin prick tests are used to confirm the diagnosis [66].

### Implications for practice

TXA has gained widespread use as an effective means of promoting haemostasis and reducing intraoperative blood loss in orthopaedic surgery. However, the best practices for TXA are still unclear. Our study reveals peri-articular injection as an alternative route for TXA injection. In addition, we recommended using TXA less than 1.5 g, and to further reduce blood loss, PAI can be combined with IV and IA.

### Implications for clinical research

As PAI is a new injection route, we suggest the following investigation guidelines to help further discussions. First, include patients who are undergoing joint arthroplasty, such as hip and shoulder arthroplasty. Arthroscopic surgery and trauma surgery should also be considered. Second, interventions need to consider PAI combined with oral, IV, or IA TXA. Comparisons need to consider IV combined with IA or oral TXA, or different doses of PAI TXA. Outcomes should include ecchymosis, haematoma formation, VAS (visual analogue scale) score, and subjective joint function scores. In addition, the application of tourniquet in perioperative injection of TXA in knee surgery is also worth considering. Finally, more RCTs are needed.

### Limitations

There are several limitations to this study. First, as a newly mentioned TXA administration, PAI has been studied by few RCTs, and the inclusion of non-RCT studies made our data less valid than ideal. Second, in the comparison between PAI and IA or IV, the TXA dose in the two groups in some studies was inconsistent, which may have affected the results. In addition, TXA was mixed with other drugs during injection, which may mask some effects. Third, in the process of data synthesis, different units were used for the same outcome index, resulting in some data not being included. Finally, we could not evaluate the specific optimal dose of TXA for PAI, only deducing that TXA less than 1.5 g may be more suitable. More importantly, PAI in combination with IV or IA could not be further compared to the combination of IV and IA.

## Conclusion

PAI has comparable effects to those of IV and IA injections. PAI is an alternative injection route of TXA administration for patients who have undergone TKA.

## Data Availability

The present study was a review of the previously published literature.

## Abbreviations

TXA: Tranexamic acid
IV: Intravenous
IA: Intra-articular
TKA: Total knee arthroplasty
PAI: Peri-articular injection
PAMC: Peri-articular multimodal cocktail
PT: Prothrombin time
APTT: Activated partial thromboplastin time
NOS: Newcastle–Ottawa Scale
MCMS: Modified Coleman Methodology Score
RCTs: Randomized controlled trials
PRISMA: Preferred Reporting Items for Systematic Reviews and Meta-Analyses
CI: Confidence interval
MD: Mean difference
OR: Odds ratios
